# The Economy of Bacteria

**Published:** 1883-01

**Authors:** J. M. Adams


### THE ECONOMY OF BACTERIA.

BY J. M. ADAMS.

In the study of bacteria my attention has been specially called to their exceedingly numerous existence in all the waste matters of the human system, and much more so than generally supposed. In urine, pus, serum, etc., we are all generally acquainted with the well-known supply of these organisms as soon as oxidation commences. After many examinations, I have not yet found a single specimen of human excrement, either from young or old, that did not contain astonishingly vast numbers of full grown bacteria. This is the most prolific source of supply I have yet seen. The food, after digestion and absorption by the smaller intestines, commences to decompose, and before its passage the bacteria spring into life in countless myriads and verily swarm in every part of the fæces.

To be seen well, the material must be greatly reduced with water and a strong clear light used with a power of not less than five hundred or, better, a thousand or more diameters.

It is evident there could not be such a rapid outgrowth without the previous existence of germs or spores in all kinds of food—both animal and vegetable.

The microscope, strange to say, is not really able to detect the very minute spores of the smaller forms; but by comparison with the larger forms of bacteria, it is reasonable to infer that they really do exist, although unseen, and that these spores may have been formed at a previous decomposition of the substance and preserved through growth till a subsequent decomposition of the same.

The inference is pretty conclusive that the germs of bacteria never wholly pass out of existence in any organized product, and that by nature they are designed to exist through all ordinary changes of surrounding life.

The difference in the forms of the smallest and lowest species, although somewhat similar, show that several species generally exist together, and often very many curious and varied forms are found in decomposing nitrogenous substances.

There also appear to be many larger forms of a higher order, much more diversified in shape (which are not an enlargement of the lower) found in older and more putrid products. These seem to require a stronger and richer soil as it were, where all is favorable for their growth, provided, however, that the germs exist in the substance, or become in any way lodged in it.

It may be stated also, that the decay of organized material is always attended at certain stages, with bacteria; that they convert the bioplasm or protoplasm into crude elements for newly organized material. They require oxygen, carbon, nitrogen, and some mineral salts, for growth. Those species connected with "fermentation" must have more oxygen; those "giving color," more carbon; those "producing disease," more nitrogen. It may be possible that the same species may produce different results in different materials, yet they seem to have their specific work, and do it rapidly.

The same relations and results exist with the more minute micrococci—a still lower form of organization than the usual elongated bacterian forms—being mere points and almost invisible under the highest powers.

These latter seem to be more attendant with disease than the other, and their agency is certainly very important, being connected with most all of the inflammatory and irritant diseases, and directly disturbing the vital functions.

The ordinary bacteria are mainly innocent in themselves or simply indicative of disease and decomposition. They may become impregnated with poisonous volatile alkaloids which some species produce, and thus communicate or produce disease, but their work is to tear down the organized structure and decompose it.

They are not to be condemned altogether, for very great good is done by them in their proper work by aiding and hastening decomposition beyond that which oxidation could do alone, but with oxidation produce a ten fold activity.

The worst forms are not more malignant or to be dreaded than certain minute fungi, with their greedy spores and exhaustive mycelium, one of which was so well and truly described by Dr. Blackham, in the October number of the BISTOURY.

